# How the epidemiology of disease-resistant and disease-tolerant varieties affects grower behaviour

**DOI:** 10.1098/rsif.2022.0517

**Published:** 2022-10-19

**Authors:** Rachel E. Murray-Watson, Nik J. Cunniffe

**Affiliations:** Department of Plant Sciences, University of Cambridge, Cambridge CB2 1TN, UK

**Keywords:** behavioural model, epidemiological model, resistance, tolerance, tomato yellow leaf curl virus

## Abstract

Population-scale effects of resistant or tolerant crop varieties have received little consideration from epidemiologists. When growers deploy tolerant crop, population-scale disease pressures are often unaffected. This only benefits growers using tolerant varieties, selfishly decreasing yields for others. However, resistant crop can reduce disease pressure for all. We coupled an epidemiological model with game theory to understand how this affects uptake of control. Each time a grower plants a new crop, they must decide whether to use an improved (i.e. tolerant/resistant) or unimproved variety. This decision is based on strategic-adaptive expectations in our model, with growers comparing last season’s profit with an estimate of what is expected from the alternative crop. Despite the positive feedback loop promoting use of a tolerant variety whenever it is available, a mixed unimproved- and tolerant-crop equilibrium can persist. Tolerant crop can also induce bistability between a scenario in which all growers use tolerant crop and the disease-free equilibrium, where no growers do. However, due to ‘free-riding’ by growers of unimproved crop, resistant crop nearly always exists in a mixed equilibrium. This work highlights how growers respond to contrasting incentives caused by tolerant and resistant varieties, and the distinct effects on yields and population-scale deployment.

## 1. Introduction

Tolerance and resistance represent the two main mechanisms underpinning the genetic control of plant disease, and the differences between the two have clear implications for epidemic management. Resistance traits are associated with a reduction in pathogen burden [1], and disease-resistant varieties are consequently less susceptible and/or infectious than unimproved varieties. By contrast, tolerant varieties can be infected and maintain high pathogen burdens, meaning that infected plants can transmit infection at high rates, but their yield remains high in comparison with unimproved crop [1]. The two traits can be difficult to distinguish during breeding programmes, as both tolerance and resistance characteristics preserve yields when a plant is infected [2]. Partial or quantitative resistance where the host does not completely restrict viral load but has a lower yield loss [3,4] is also more common than complete (qualitative) immunity [5], further complicating the distinction between tolerance and resistance in breeding.

Epidemiological models have long been used to identify strategies that optimize the deployment of resistant crop (reviewed in [6]). Very often the focus is on pathogen evolutionary dynamics and the breakdown of resistance traits [7–10]. This builds on a long history of models aiming to explain gene-for-gene polymorphisms in host and pathogen populations, stretching back to theoretical work, which is now nearly 50 years old [11] although it is still of current interest [12–14]. However, no studies have compared the epidemiological consequences of using tolerant versus resistant crop at the population scale, despite tolerant and resistant crop having significantly distinct effects on other growers. There has also been no consideration of factors incentivizing growers to deploy one or other of these possible disease controls.

However, no studies to date have compared the epidemiological consequences of using tolerant vs resistant crop. Recent models have also incorporated pathogen evolutionary dynamics and the breakdown of resistance traits. However, in our study, we only consider the epidemiological, not evolutionary, effects of the use of resistant or tolerant crop.

In economics, externalities are the effect of an action by one party on other parties [15]. The previous work has shown that in disease management schemes, prevention measures such as vaccinations generate enough *positive externalities* that they disincentivize others from partaking in control [16,17]. By lowering the infection burden, this type of control increases the probability that those not using the control scheme will be ‘successful free-riders’ (i.e. gain the benefit of control without paying any of the costs [18]). In plant epidemiology, resistance traits lower the prevalence of infection in the system as a whole and thus have the same impact as vaccinations by decreasing the probability of infection for non-resistant crop. Previous theoretical studies have shown that not every grower need plant-resistant crop for the benefits to be felt across a community of growers [9,19,20]. This same result applies to other mechanisms of control. Although high participation in citrus health management areas (voluntary schemes established in the United States to combat citrus greening via methods such as synchronous spraying of pesticides) is correlated with better outcomes, complete participation is not needed to see improved yield outcomes [21].

Similarly, the majority of the benefits conferred by *Bt*-resistant maize in the United States was experienced by those who did not themselves use the improved crop and were consequently ‘free-riding’ off the actions of others [22]. Reducing the degree of resistance could diminish these positive externalities, but ultimately some growers planting resistant varieties still acts as a disincentive to other individuals to practise disease management.

Disease tolerance, by contrast, should have the opposite effect to resistance. Tolerant crops do not restrict pathogen replication [1,23] and so do not reduce the probability of infection for others. Tolerant crops, however, can sustain such pathogen burdens without the same degree of yield loss as unimproved crops [1]. As tolerant crops have less noticeable symptoms, they cannot be as effectively removed and/or treated for disease. Thus, growers who use tolerant crops will experience the benefit whilst generating *negative* externalities for those around them by maintaining a high infection pressure [24]. This effect may weaken other disease management efforts, increasing the negative consequences for others (see [25] for an example in human disease epidemiology where it was found that tolerance-based therapies for chronic infections increase population-level mortality as asymptomatic carriers circulate in the population). Tolerant crops may be asymptomatic and thus have a reduced probability of being visually detected and rogued (removed) in comparison with unimproved crops [26], again allowing an increase in infection pressure. This incentivizes others to use tolerant crops too, to minimize their losses, and could lead to overall higher participation in control schemes.

Game theory is an economic tool used to examine strategic decision-making among interacting parties [27]. An individual’s response to the threat of disease is highly contingent on the actions of others. For example, an individual’s choice to vaccinate may depend on how many others in the population have been vaccinated (e.g. [18,28]). Game theory has been increasingly used to better understand some of the drivers of human behaviour in response to an epidemic [29]. Models derived from game-theoretic principals have also been used to examine the behaviour of growers who are making decisions about crop management [30–33]. Here, we use a game-theoretic model to examine the effects of the contrasting externalities of tolerant and resistant crop.

To examine the differing effects of disease tolerance and resistance on the profits and, consequently, the behaviour of growers, we employ *tomato yellow leaf curl virus* (TYLCV) as a case study. Tomato (*Lycopersicon esculentum*) is a globally important crop, with over 18.5 million tonnes produced in 2020 alone [34]. TYLCV is one of the major viruses affecting tomato production worldwide [35], and it has been detected from East Asia to Western Europe, the United States of America and Australia. Transmitted by the sweet potato whitefly *Bemisia tabaci* [36], infection leads to tomato yellow leaf curl disease (TYLCD), which causes curling of the leaves, chlorosis of young leaves, flower abortion and stunting. Combined, these symptoms can cause up to 100% yield loss. Consequently, much research has been done on developing both tolerant and resistant varieties [37,38], some of which have been deployed [39,40].

Despite the expansive literature, and perhaps reflecting the confusion between tolerance and resistance across crop breeding, there is considerable debate as to what degree of disease resistance has been achieved in tomato breeding. Some argue that though purportedly TYLCV-‘resistant’ cultivars have been developed, none have complete immunity to infection ([4,41] describe how what they term ‘resistant lines’ are not actually immune to TYLCV, but instead have reduced symptoms). As there is incomplete restriction of viral replication, yield loss is less extreme than in completely susceptible genotypes. Some disease-resistant accessions have been identified in wild relatives of tomato [41], and others have produced what appear to be genuinely disease-resistant varieties of tomato [38], which will possibly lead to the deployment of fully resistant cultivars in the future.

Additional complications such as the dependence of symptom development on non-genetic factors such as when in its life cycle the host was infected [42], or even which part of the plant was inoculated [43] again make it harder to distinguish between resistance and tolerance. However, even the incomplete restriction of viral replication means that these cultivars can be considered partially resistant, rather than tolerant. To account for the difficulties in establishing whether a cultivar is tolerant or resistant, we consider a range of parameters pertaining to both qualities. These parameters represent a continuum between tolerance and resistance characteristics, allowing us to move smoothly from one parametrization to another.

We use this case study to investigate the following questions: (1) How does the average profit of a group of growers change when a fixed proportion of those growers are using crop that is either tolerant or resistant to disease? (2) When growers can choose which type of crop they plant, how do the initial proportion of infectious and controlled fields affect the deployment of tolerant or resistant crop? (3) How does the use of improved crop change depending on whether it is disease tolerant or resistant?

## 2. Description

In our model of disease spread amongst a system of tomato fields, there are two available crop varieties: unimproved crop (*U*, ‘uncontrolled’ or ‘unimproved’) or an improved variety (*C*, ‘controlled’). The latter has some degree of tolerance and/or resistance depending on the scaling of certain epidemiological and economic parameters. We can break down this tolerance/resistance continuum into six parameters (table 1), which relate to how TYLCV is transmitted and the losses sustained when a field is infectious.

**Table 1.** Parameters related to resistant and tolerant varieties. The distinction between the two varieties lies in how disease is transmitted and what losses are incurred when a field is infectious.

parameter	meaning	value if resistant	value if tolerant
*δ*_*β*_	relative susceptibility of improved variety	0.5	1
*δ*_*σ*_	relative infectivity of improved variety	0.5	1
*δ*_*Y*_	relative yield of improved variety	1	1
*δ*_*L*_	relative losses due to disease of improved variety	1	0.1
1/*δ*_*ε*_	relative latent period for improved crop	0.5	1
*δ*_*ν*_	relative probability of detection for improved crop	1	0.1

We classify fields based on their infectious states (susceptible, *S*; latently infected and asymptomatic, *E*; or infectious and symptomatic, *I*) and by the variety with which they are planted (subscripts *U* and *C*). We do not model within-field disease spread and assume that the two control strategies are mutually exclusive: a field cannot be planted with both unimproved and improved crop.

Susceptible fields are infected with TYLCV (transmitted by viruliferous *B. tabaci*) at rate *β* or *δ*_*β*_*β* for unimproved and improved crops, respectively. Once infected (and provided they are not first harvested), fields remain latently infected (i.e. are asymptomatic and cannot transmit disease) for an average of 1/*ε*, days, after which they become infectious. Crop that is resistant to infection has an extended latent period, as symptoms take longer to develop. The latent period is increased by a factor of $\delta_{\epsilon_{R}}<1$ (ensuring that $\delta_{\epsilon_{R}}\epsilon <\epsilon$). Fields are harvested on average every 1/*γ* days, irrespective of their control type or infection status.

By restricting within-host viral replication, resistant crop also has a reduced probability of transmitting disease. The relative infectivity of a resistant field is given by $\delta_{\sigma }$.

We also add roguing as a control mechanism enacted by all growers irrespective of the crop variety they use. Roguing infected plants is a common practice for management of TYLCV [44–47]. In line with our assumption regarding symptom emergence and infectivity, only infectious crops (*I*_*i*_, *i* ∈ {*U*, *C*}) are rogued. Visual scouting for infection occurs at time intervals of Δ.

Tomato is a climacteric fruit, meaning it can ripen once harvested from the plant [48]. Growers, therefore, who harvest before ‘full maturity’ call still gain marketable product [48], though there will be a yield penalty for early harvest as immature fruit have inferior flavours and are more susceptible to damage [49]. As this penalty is less than the loss due to disease, growers who detect infection in their fields will do better by prematurely harvesting all crop to prevent disease progression (reducing losses by a factor *ϕ*_*R*_).

The degree of symptom severity will differ between unimproved and improved crop. As such, each crop type will have its own probability of detection (*ν* and *δ*_*ν*_*ν*). We presume that when the improved crop has ‘tolerant’ characteristics, the milder symptoms result in a low probability of detection (i.e. *δ*_*ν*_ < 1; table 1). However, if the improved crop has ‘resistance’ characteristics but nevertheless becomes infected, it is detected with the same probability as the unimproved crop (i.e. *δ*_*ν*_ = 1). The average length of time to detect an infectious field is therefore given by Δ/*ε* and $\Delta /(\delta_{\epsilon }\epsilon )$

The emergence of symptoms occurs at a constant rate (1/*ε* or $1/\delta_{\epsilon }\epsilon$ ) and can therefore occur at any time between the previous round of surveys (when the field was asymptomatic) and the following round (when the field is symptomatic). Infectious fields will, on average, be symptomatic for half of this time period (Δ/2).

Unimproved and controlled fields are removed at rates *μ*_*U*_ and *μ*_*C*_, respectively, which are given by [50]

2.1 $$\mu_{U}=\frac{1}{((1/\nu )-(1/2))\;\Delta }$$

and

2.2 $$\mu_{C}=\frac{1}{((1/\delta_{\nu }\nu )-(1/2))\;\Delta }.$$

As we are modelling on the scale of a field, we presume that if a field is rogued, it is then replanted immediately with healthy crop. The epidemiological model is given by

2.3 $$\frac{dS_{C}}{dt}=\gamma C+\mu_{C}I_{C}-\delta_{\beta }\beta S_{C}(I_{U}+\delta_{\sigma }I_{C})-\gamma S_{C},$$

2.4 $$\frac{dE_{C}}{dt}=\delta_{\beta }S_{C}(I_{U}+\delta_{\sigma }I_{C})-\delta_{\epsilon }\epsilon E_{C}-\gamma E_{C},$$

2.5 $$\frac{dI_{C}}{dt}=\delta_{\epsilon }\epsilon E_{C}-\mu_{C}I_{C}-\gamma I_{C},$$

2.6 $$\frac{dS_{U}}{dt}=\gamma U+\mu_{U}I_{U}-\beta S_{U}(I_{U}+\delta_{\sigma }I_{C})-\gamma S_{U},$$

2.7 $$\frac{dE_{U}}{dt}=\beta S_{U}(I_{U}+\delta_{\sigma }I_{C})-\epsilon E_{U}-\gamma E_{U}$$

and

2.8 $$\;\frac{dI_{U}}{dt}=\epsilon E_{U}-\mu_{U}I_{U}-\gamma I_{U},$$

where *C* = *S*_*C*_ + *E*_*C*_ + *I*_*C*_ and *U* = *S*_*U*_ + *E*_*U*_ + *I*_*U*_.

The rate of roguing applies to the field level, but when we calculate the profits for each strategy we do so for individual growers. Roguing aims to minimize yield loss; the losses due to disease (*L*) are thus reduced by a factor *ϕ*_*R*_ < 1 that represents the relative benefit of harvesting an infected field before the end of the season and thus avoiding the maximum yield loss. We assume that roguing and replanting of a rogued field occur instantaneously. The benefit provided by roguing appears in the profits of those growers that rogued an infectious field.

Parameters for this model are summarized in table 2, and initial conditions are outlined in table 3.

**Table 2.** Summary of parameter values. When the parameter relates to improved crop, the first value is for tolerant crop and the second for resistant.

parameter	meaning	value	reference
1/*γ*	length of the growing season	120 d	[51,52]
*β*	rate of secondary infection	0.055/N d^−1^ field^−1^	see main text
Δ	time between roguing	120 d	illustrative
*ν*	probability of detection	1	illustrative
*μ*_*U*_	removal rate (unimproved)	1/60 d^−1^	illustrative
*μ*_*C*_	removal rate (improved)	1/60 or 1/1140 d^−1^	illustrative
1/*ε*	average latent period	41 d	[43,53]
*η*	responsiveness of growers	10	[32]
*Y*	maximum yield	1	all values scaled relative to yield
*L*	loss due to infection	0.6	[40]
*ϕ*_*C*_	cost of improved crop	0.1	[54]
*ϕ*_*R*_	relative reduction in loss due to roguing	0.7	illustrative
*N*	total number of fields/growers	1	scaled to 1

**Table 3.** Default initial conditions. These are scaled to be proportions (i.e. *N* = 1).

variable	meaning	value
*S*_*C*_(0)	initial proportion of susceptible controlling fields	0.1 N
*E*_*C*_(0)	initial proportion of latently infected controlling fields	0 N
*I*_*C*_(0)	initial proportion of infectious controlling fields	0 N
*S*_*U*_(0)	initial proportion of susceptible non-controllers	0.89 N
*E*_*U*_(0)	initial proportion of latently infected non-controlling fields	0 N
*I*_*U*_(0)	initial proportion of infectious non-controllers	0.01 N

### 2.1. Parametrization

Where possible, parameter values were taken from the literature. A summary of the parametrization is given in table 2, with full details discussed in electronic supplementary material, appendix 2.

### 2.2. Growers’ profits

To determine the benefits provided by each strategy, we estimate the expected profits of a grower using a particular strategy. These are then compared with the grower’s profit from the previous season to determine if the grower should consider switching strategy. These profits account for the costs and losses associated with each crop type and infection outcome and are given by

2.9 $$P_{\mathrm{SU}}=\text{Profit for non-controller with a susceptible field}=Y,\;$$

2.10 $$P_{\mathrm{EU}}=\text{Profit for non-controller with latently infected field}=Y,$$

2.11 $$\begin{array}{rl} P_{\mathrm{IUH}} & =\text{Profit for non-controller with an infected field that}\\ & \;\text{was not rogued}=Y-L, \end{array}$$

2.12 $$\begin{array}{rl} P_{\mathrm{IUR}} & =\text{Profit for non-controller with an infected field}\\ & \;\text{that was rogued}=Y-\phi_{R}L, \end{array}$$

2.13 $$P_{\mathrm{SC}}=\text{Profit for controller with a susceptible field}=Y-\phi_{C},$$

2.14 $$P_{\mathrm{EC}}=\text{Profit for controller with latently infected field}=Y-\phi_{C},$$

2.15 $$\begin{array}{c} P_{\mathrm{ICH}}=\text{ Profit for controller with an infected field}\;\\ \;\;\text{ that was not rogued}=Y-\phi_{C}-\delta_{L}L,\; \end{array}$$

2.16 $$\begin{array}{c} P_{\mathrm{ICR}}=\text{ Profit for controller with an infected field and}\;\\ \text{ that was rogued}=Y-\phi_{C}-\phi_{R}\delta_{L}L.\; \end{array}$$

The profit for an uninfected or latently infected field using unimproved crop (*P*_SU_ or *P*_EU_) will always the maximum achievable profit, as at the time of harvest these growers have avoided paying the cost of control or incurring any losses due to infection. We differentiate between profits of growers with infectious fields by whether their field was rogued before harvesting and thus had a lower yield loss (*ϕ*_*R*_*L* or *ϕ*_*R*_*δ*_*L*_*L*).

As we only consider the case where the costs of control are less than the losses due to disease in unimproved crop (*ϕ*_*C*_ < *L*), the relative sizes of the remaining profits depend on the tolerance/resistance characteristics of the improved crop. Under the default parametrization provided in tables 1 and 2, tolerant crop, where *δ*_*L*_ *L* < *L*, the order of profits is

2.17 $$P_{\mathrm{SU}}=P_{\mathrm{EU}}>P_{\mathrm{SC}}=P_{\mathrm{EC}}>P_{\mathrm{ICR}}>P_{\mathrm{ICH}}>P_{\mathrm{IUR}}>P_{\mathrm{IUH}}$$

and for the default resistant background is, where *δ*_*L*_*L* = *L*

2.18 $$P_{\mathrm{SU}}=P_{\mathrm{EU}}>P_{\mathrm{SC}}=P_{\mathrm{EC}}>P_{\mathrm{IUR}}>P_{\mathrm{IUH}}>P_{\mathrm{ICR}}>P_{\mathrm{ICH}}.$$

A full explanation of these orderings is found in electronic supplementary material, appendix 2.

In the case where there is a fixed proportion of growers using each strategy, we can quantify the benefit each strategy provides by calculating the average profits for a grower using each crop type. To do this, we must first define the probability that an infectious field has not been rogued (*q*_IUH_ and *q*_ICH_ for unimproved and improved fields, respectively),

2.19 $$q_{\mathrm{IUH}|I_{U}}=\frac{\gamma }{\gamma +\mu_{U}}$$

and

2.20 $$q_{\mathrm{ICH}|I_{C}}=\frac{\gamma }{\gamma +\mu_{C}},$$

and the field has been rogued, which is expressed as follows:

2.21 $$q_{\mathrm{IUR}|I_{U}}=\frac{\mu_{U}}{\gamma +\mu_{U}}$$

and

2.22 $$q_{\mathrm{ICR}|I_{C}}=\frac{\mu_{C}}{\gamma +\mu_{C}}.$$

The average profits for unimproved (*P*_*U*_) and improved (*P*_*C*_) crop are given by:

2.23 $$P_{U}=\frac{S_{U}P_{\mathrm{SU}}+E_{U}P_{\mathrm{EU}}+(\gamma /\left(\mu_{U}+\gamma \right))I_{U}P_{\mathrm{IUH}}+(\mu_{U}/\left(\mu_{U}+\gamma \right))I_{U}P_{\mathrm{IUR}}}{U},$$

2.24 $$P_{C}=\frac{S_{C}P_{\mathrm{SC}}+E_{C}P_{\mathrm{EC}}+(\gamma /\left(\mu_{C}+\gamma \right))I_{C}P_{\mathrm{ICH}}+(\mu_{C}/\left(\mu_{C}+\gamma \right))I_{C}P_{\mathrm{ICR}}}{C},$$

where *U* = *S*_*U*_ + *E*_*U*_ + *I*_*U*_ and *C* = *S*_*C*_ + *E*_*C*_ + *I*_*C*_.

### 2.3. Calculating expected profits

As mentioned earlier, growers were assigned a control strategy at the beginning of the epidemic and could not change to the alternative, irrespective of profitability or grower preference. However, it is expected that growers will instead choose whether to control based on the perceived profitability of control, which will depend on parameters such as the cost of control and also the current risk of infection.

We use the ‘grower vs alternative’ mechanism for decision-making, as set out in [30–33]. In this behavioural model, growers compare their outcome from the previous season with the expected profit of the alternative strategy (i.e. the strategy that they did not previously adopt), which in turn is based on the instantaneous probability of infection. The probability that they change strategy is then based on the magnitude of the differences between their previous profit and the profit of the alternative strategy.

The full mathematical derivation of the expected profits is found in electronic supplementary material, appendix 1. The expected profits for a non-controller, *P*_*U*_ is therefore given by

2.25 $$P_{U}=Y-q_{U}\frac{\epsilon }{\epsilon +\gamma }L\left(\frac{\gamma }{\gamma +\mu_{U}}+\frac{\mu_{U}}{\mu_{U}+\gamma }\phi_{R}\right)\;$$

and

2.26 $$P_{C}=Y-\phi_{C}-q_{C}\frac{\delta_{\epsilon }\epsilon }{\delta_{\epsilon }\epsilon +\gamma }\delta_{L}L\left(\frac{\gamma }{\gamma +\mu_{C}}+\frac{\mu_{C}}{\mu_{C}+\gamma }\phi_{R}\right),$$

where *q*_*U*_ is the probability of infection for an uncontrolled field $\left(\left(\beta (I_{U}+\delta_{\sigma }I_{C})\right)/\left(\beta (I_{U}+\delta_{\sigma }I_{C})+\gamma \right)\right)$ and *q*_*C*_ is the probability of infection for a controlled field $\left(\left(\delta_{\beta }\beta (I_{U}+\delta_{\sigma }I_{C})\right)/\left(\delta_{\beta }\beta (I_{U}+\delta_{\sigma }I_{C})+\gamma \right)\right)$ (electronic supplementary material, appendix 1).

### 2.4. Switching terms based on the expected profits

From equations (2.25) and (2.26), we can base determine the probability of a grower of outcome *i* ∈ {*S*_*U*_, *E*_*U*_, *I*_*U*_, *S*_*C*_, *E*_*C*_, *I*_*C*_} switching into the alternative strategy [32]. These ‘switching terms’ compare the difference between the grower’s profit from the previous season with the expected profit of the strategy that they did not adopt. These differences are multiplied by a ‘responsiveness’ parameter, *η*, which accounts for the responsiveness of growers to differences in profit [32]. If the expected profit is less than the grower’s current profit, the grower should not switch strategy. The payoff for a non-controller that harvests susceptible crop (*P*_SU_ and, for the default parametrization, *P*_EU_ from latently infected fields) should always be the highest as they do not pay the cost of control or losses due to disease. These growers should therefore never switch strategy. Which is the lowest payoff will depend on the strength of the tolerance or resistance traits in the improved crop, as well as the relative costs (equations (2.17) and (2.18); electronic supplementary material, appendix 2). If it is tolerant, the loss for tolerant crop will be less than that of unimproved crop (*δ*_*L*_ *L* < *L*). Consequently, *P*_IUR_ is the lowest payoff. Conversely, if the improved crop is resistant, *δ*_*L*_ *L* = *L* and the cost of control means that *P*_ICR_ is the lowest payoff.

The switching terms are given by

2.27 $$z_{\mathrm{SU}}=\max(0,1-e^{-\eta (P_{C}-P_{\mathrm{SU}})}),$$

2.28 $$z_{\mathrm{EU}}=\max(0,1-e^{-\eta (P_{C}-P_{\mathrm{EU}})}),$$

2.29 $$z_{\mathrm{IUH}}=\max(0,1-e^{-\eta (P_{C}-P_{\mathrm{IUH}})}),$$

2.30 $$z_{\mathrm{IUR}}=\max(0,1-e^{-\eta (P_{C}-P_{\mathrm{IUR}})}),$$

2.31 $$z_{\mathrm{SC}}=\max(0,1-e^{-\eta (P_{U}-P_{\mathrm{SC}})}),$$

2.32 $$z_{\mathrm{EC}}=\max(0,1-e^{-\eta (P_{U}-P_{\mathrm{EC}})}),$$

2.33 $$z_{\mathrm{ICH}}=\max(0,1-e^{-\eta (P_{U}-P_{\mathrm{ICH}})})$$

2.34 $$\mathrm{and}\;\;\;z_{\mathrm{ICR}}=\max(0,1-e^{-\eta (P_{U}-P_{\mathrm{ICR}})}).$$

Incorporating these into the epidemiological model, we have

2.35 $$\frac{dS_{C}}{dt}=\gamma \theta_{C}-\delta_{\beta }\beta S_{C}(I_{U}+\delta_{\sigma }I_{C})+M_{C}-\gamma S_{C},$$

2.36 $$\frac{dE_{C}}{dt}=\delta_{\beta }\beta S_{C}(I_{U}+\delta_{\sigma }I_{C})-\delta_{\epsilon }\epsilon E_{C}-\gamma E_{C},$$

2.37 $$\frac{dI_{C}}{dt}=\delta_{\epsilon }\epsilon E_{C}-\mu_{C}I_{C}-\gamma I_{C},$$

2.38 $$\frac{dS_{U}}{dt}=\gamma \theta_{U}-\beta S_{U}(I_{U}+\delta_{\sigma }I_{C})+M_{U}-\gamma S_{U},$$

2.39 $$\frac{dE_{U}}{dt}=\beta S_{U}(I_{U}+\delta_{\sigma }I_{C})-\epsilon E_{U}-\gamma E_{U}$$

2.40 $$\mathrm{and}\;\frac{dI_{U}}{dt}=\epsilon E_{U}-\mu_{U}I_{U}-\gamma I_{U},$$

where

2.41 $$\theta_{C}=(1-z_{\mathrm{SC}})S_{C}+(1-z_{\mathrm{EC}})E_{C}+(1-z_{\mathrm{ICH}})I_{C}+z_{\mathrm{IUH}}I_{U},$$

2.42 $$\theta_{U}=S_{U}+(1-z_{\mathrm{EU}})E_{U}+(1-z_{\mathrm{IUH}})I_{U}+z_{\mathrm{SC}}S_{C}+z_{\mathrm{EC}}E_{C}+z_{\mathrm{ICH}}I_{C},$$

2.43 $$M_{C}=(1-z_{\mathrm{ICR}})\mu_{C}I_{C}+z_{\mathrm{IUR}}\mu_{U}I_{U},$$

2.44 $$M_{U}=z_{\mathrm{ICR}}\mu_{C}I_{C}+(1-z_{\mathrm{IUR}})\mu_{U}I_{U}$$

2.45 $$\mathrm{and}\;N=S_{C}+E_{C}+I_{C}+S_{U}+E_{U}+I_{U}.$$

**Figure 1.**
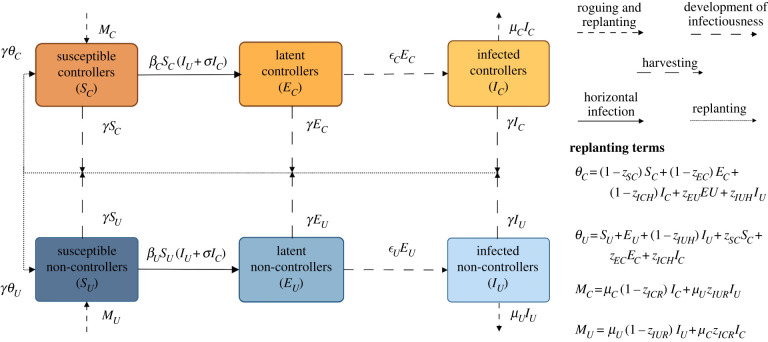
Schematic showing the structure of the model when growers can choose their strategy based on expected profits. We have two classes of grower, those who use unimproved seed (*U*) and those who use improved seed (*C*). This improved seed comes in one of the two varieties: tolerant or resistant. The terms *θ*_*C*_ and *θ*_*U*_ are the rates of replanting for harvested improved and unimproved fields, whilst *M*_*C*_ and *M*_*U*_ are rates of replanting for rogued fields (equations (2.41)–(2.44), with the total replanting rate of rogued fields given by: *M*_*C*_ + *M*_*U*_ = *μ*_*C*_ *I*_*C*_ + *μ*_*U*_ *I*_*U*_). Created with BioRender.com.

We highlight here that in equation (2.42), *S*_*U*_ and *E*_*U*_ are not associated with any switching terms as for our parametrization, and the values of *z*_SU_ and *z*_EU_ are zero. A schematic of the model is presented in figure 1.

Which switching terms are positive or fixed at zero depends on the parameter values and the epidemiological state of the system. Due to the ordering of the payoffs, only certain combinations of positive switching terms are possible (electronic supplementary material, appendix 2).

## 3. Results

### 3.1. Q1: Externalities of tolerant and resistant crop with fixed proportions of growers

#### 3.1.1. Basic reproduction number, *R*_0_

When there is only one type of crop (improved, which can be either resistant or tolerant, or unimproved), the basic reproduction numbers are as follows:

3.1 $$R_{0U}=\frac{\beta \epsilon N}{\left(\mu_{U}+\gamma )(\epsilon +\gamma \right)}$$

and

3.2 $$R_{0C}=\frac{\delta_{\beta }\beta \delta_{\epsilon }\epsilon \delta_{\sigma }N}{\left(\mu_{C}+\gamma )(\delta_{\epsilon }\epsilon +\gamma \right)},$$

where for *R*_0*U*_, *N* = *U* and for *R*_0*C*_, *N* = *C*. In these expressions, *ε*/(*μ*_*U*_ + *γ*)(*ε* + *γ*) and *δ*_*ε*_*ε*/(*μ*_*C*_ + *γ*)(*ε* + *γ*) are the probabilities that a field will become infectious before it is harvested for unimproved and controlled fields respectively. The mean time spent in the *I*_*i*_ compartment is 1/(*μ*_*i*_ + *γ*) (with *i* ∈ {*U*, *C*}) the number of infections given caused by these infectious fields is *βU* or *δ*_*β*_*βC* for unimproved or improved, respectively. The value of *R*_0*C*_ also accounts for the reduced infectivity of infectious improved crop (*δ*_*σ*_).

We use the next-generation matrix (NGM) method [55] to evaluate *R*_0_ when both crop types are present at the disease-free equilibrium (DFE) (i.e. (*S*_*U*_, *E*_*U*_, *I*_*U*_, *S*_*C*_, *E*_*C*_, *I*_*C*_) = (*U*, 0, 0, *C*, 0, 0)) (see electronic supplementary material, appendix 3).

*R*_0_, when both crop types are present, is given by

3.3 $$\begin{array}{rl} R_{0} & =\frac{U}{N}R_{0U}+\frac{C}{N}R_{0C},\\ & =\frac{\beta \epsilon U}{\left(\gamma +\mu_{U})(\gamma +\epsilon \right)}+\frac{\delta_{\beta }\beta \delta_{\epsilon }\epsilon \delta_{\sigma }C}{(\gamma +\mu_{C})(\gamma +\epsilon ).} \end{array}$$

This is a combination of equations (3.1) and (3.2), scaled in proportion with the proportion using each crop type [56,57].

#### 3.1.2. Effect of changing proportion of improved crop

**Figure 2.**
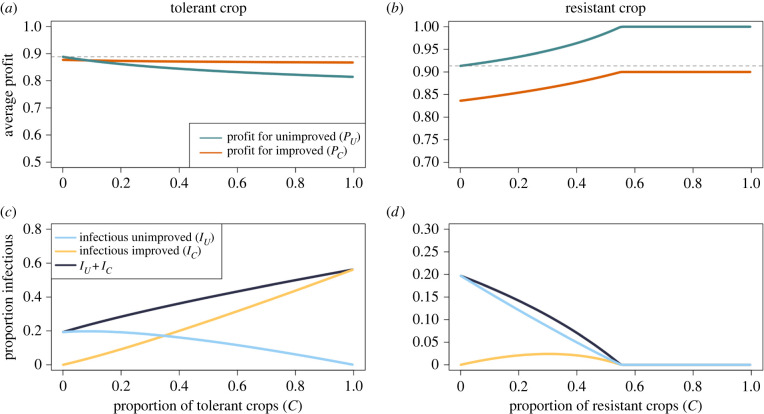
Effect of increasing proportions of improved crop on the average profit of growers using unimproved and improved crop. (*a*,*c*) Use the tolerant paramet rization, whilst (*b*,*d*) show the same for resistant crops. The two types of improved crop had opposite effects; as the proportion of improved, tolerant crop increased, so too did the amount of infection. This caused a decrease in profits for non-controllers. Conversely, as *C* increased when the crop was resistant, there was a decrease in the amount of infection and concomitant increases in profits. In both cases, the grey dashed line shows the average profit of a non-controller when there are no controllers (*C* = 0), which can be used to measure the externalities generated by each type of improved crop. Parameters and initial conditions are as presented in tables 2 and 3, respectively.

We first consider the effect of an increased proportion of improved crop on the expected profits of growers of each type. By using the default parameters outlined in table 2, we can see that an increase in the proportion of growers using tolerant crops has a little impact on the expected profit of controllers (decreasing profits by approx. 1%), though reduces those of non-controllers (figure 2*a*). As tolerant crops have a lower probability of being detected (*δ*_*ν*_*ν* = 0.1) and thus a lower removal rate (*μ*_*U*_), having more tolerant crop increases the disease pressure, and fewer infectious fields are removed via roguing (figure 2*c*).

Conversely, an increase in the proportion of resistant crops provided much greater benefits to non-controllers than to controllers (figure 2*b*). Controllers already had a relatively low probability of infection, so their average profit is already close to the maximum possible (*P*_SC_ = 0.85). Thus, a decrease in the probability of infection due to an increased proportion of growers using resistant crop provides little additional benefit. With a sufficient proportion of resistant crops, disease is eliminated from the system (figure 2*d*). However, in this scenario, controllers had to continue paying the cost of control (*ϕ*_*C*_) even though there is a little need for control, so the controllers earn less than the non-controllers.

In both of these graphs, the deviation from the average profits where there is no improved crop in the system (*C* = 0, indicated by the grey dashed line) can be seen as the magnitude of the externalities generated by each crop type. As an increase in tolerant crop causes *P*_*U*_ to decrease, it generates negative externalities. Conversely, the resistant crop reduces the probability of infection and thus generates positive externalities, increasing *P*_*U*_. The increase in *P*_*U*_ at higher values of *C* (from *P*_*U*_ = 0.91 when *C* = 0 to *P*_*U*_ = 1 when *C* = 0.99) is greater than the corresponding increase in *P*_*C*_ (from *P*_*C*_ = 0.836 when *C* = 0 to *P*_*C*_ = 0.9 when *C* = 0.99), indicating that a greater benefit is felt by non-controllers than by controllers.

We have shown the above for only a single set of parameters, but the broad patterns are recapitulated for parameters controlling the effectiveness of the tolerant/resistant crop (namely, the probability of detection of improved crop (*δ*_*ν*_*ν*) and the relative susceptibility of improved crop (*δ*_*β*_) (electronic supplementary material, appendix 4, figures 1 and 2)).

### 3.2. Q2: Effect of initial conditions on long-term outcomes in the behavioural model

The complexity of the model (in particular, the presence of the switching terms) means that explicit expressions cannot be found for the values of state variables at equilibrium. However, their values and the stability of the equilibria can be evaluated numerically.

By using the NGM method (see electronic supplementary material, appendix 5), we found the basic reproduction number for the behavioural model to be

3.4 $$R_{0}=\frac{\beta \epsilon N}{\left(\mu_{U}+\gamma )(\epsilon +\gamma \right)}.$$

Broadly, this determines the stability of the DFE, as disease can only invade the system once *R*_0_ > 1. In some cases, however, bistability is possible, and there may be multiple possible equilibria below this threshold depending on the initial conditions [58].

The long-term outcomes of the model can be divided into one of four types:

— *DFE.* As *R*_0_ < 1, there are no infected fields. As there is no risk of infection, no growers use improved crop (and therefore avoid the cost of control, *ϕ*_*C*_).
— *‘No control’ equilibrium.* Disease is endemic, but no growers use improved crop.
— *‘All-control’ equilibrium.* Disease is endemic and no growers use unimproved crop.
— *Two-strategy equilibrium.* Disease is endemic and crops of both varieties are used.

For a given parameter set, it may be that two of these equilibria are locally stable, depending on the initial conditions.

**Figure 3.**
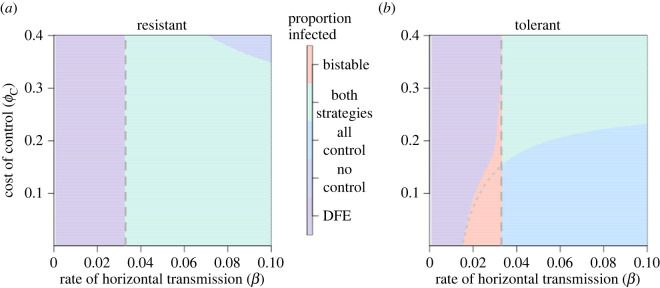
Nature of equilibria attained when improved crop is either tolerant or resistant. There are five possibilities: a DFE, with only unimproved crop; a disease-endemic, ‘no control’ equilibrium; a disease-endemic, ‘all-control’ equilibrium; and an equilibrium where both strategies and disease are present and finally a bistable region of parameter space, where two stable equilibria are possible, and so the long-term behaviour of the system depends on the initial conditions (see also figure 4). For both paramet rizations *R*_0_ = 1 is indicated by the grey vertical dashed line at *β* = 0.0333 d^−1^. (*a*) Equilibria for the resistant crop. Here, only three of the possible equilibria exist: at no point can there be an ‘all-control’ equilibrium. In addition, there is no bistable region. (*b*) Equilibria for the tolerant crop. Now, at lower costs of control and medium-to-high values of *β*, an equilibrium where all growers use improved crop is possible. There is a bistable region for 0.015 < *β* < 0.0333 d^−1^ (depending on the value of the cost, *ϕ*_*C*_). Below the dotted line in the pink bistable region, there is bistability between the ‘all-control’ and DFE; above, there is bistability between the two-strategy equilibrium and the DFE.

The parametrization of the model, both in terms of whether the improved crop is tolerant or resistant and its degree of tolerance and resistance, determines the subset of possible long-term outcomes. When the improved crop was resistant, an ‘all-control’ equilibrium was not possible (figure 3*a*). The positive externalities generated by the presence of resistant crop disincentivizes non-controllers from using improved crop, as they have a lowered probability of infection without themselves being infected. Thus, as the proportion using resistance reaches increasingly high levels, fewer non-controllers will switch to using the control scheme.

**Figure 4.**
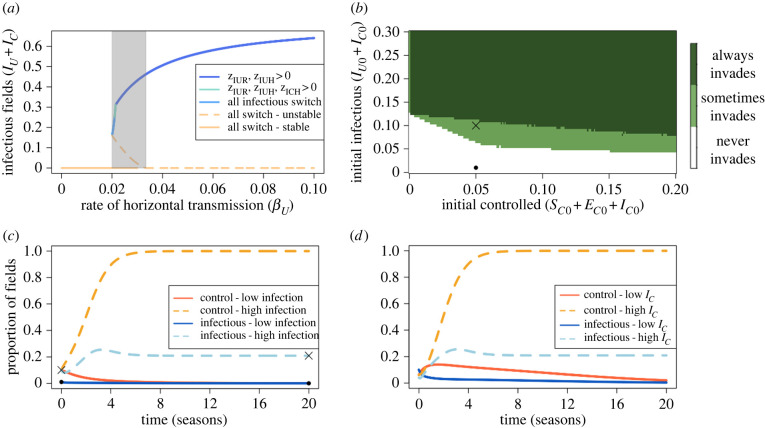
Bifurcation for the tolerant paramet rization. (*a*) Between *β* = 0.02 and 0.0333 d^−1^ (the grey region), two biologically meaningful equilibria are possible. Below *β* = 0.02 d^−1^, only the DFE is stable; above *β* = 0.0333 d^−1^ (when *R*_0_ > 1), only the disease-endemic equilibrium is. The kinks in the graph are caused by the change in the form of the switching terms. (*b*) The effect of the initial proportion of tolerant fields (*S*_*C*0_ + *E*_*C*0_ + *I*_*C*0_) and infectious fields (*I*_*U*0_ + *I*_*C*0_) on the persistence of disease at equilibrium. To only account for these factors, *E*_*U*0_ = *E*_*C*0_ = 0. When *S*_*C*0_ + *E*_*C*0_ + *I*_*C*0_ and *I*_*U*0_ + *I*_*C*0_ are very low, all initial conditions lead to the DFE. As these quantities increase, so too does the probability of disease persistence. The black cross and dot denote the proportions used in (*c*). (*c*) When *I*_*U*0_ + *I*_*C*0_ = 0.01 (‘low infectious’), disease died out. However, once *I*_*U*0_ + *I*_*C*0_ = 0.1, disease persisted. (*d*) For the ‘high infection’ paramet rization from (*c*), we investigated the effect of the proportion of infectious improved crop. When *I*_*C*0_/(*I*_*U*0_ + *I*_*C*0_) > 0.5 (as (*S*_*U*0_, *E*_*U*0_, *I*_*U*0_, *S*_*C*0_, *E*_*C*0_, *I*_*C*0_) = (0.83, 0, 0.03, 0.03, 0, 0.07); ‘high *I*_*C*_’), disease can persist. However, if *I*_*C*0_(*I*_*U*0_ + *I*_*C*0_) < 0.5 (as (*S*_*U*0_, *E*_*U*0_, *I*_*U*0_, *S*_*C*0_, *E*_*C*0_, *I*_*C*0_) = (0.83, 0, 0.07, 0.07, 0, 0.03); ‘low *I*_*C*_’), disease dies out. Other than *β* = *δ*_*β*_*β* = 0.0028 d^−1^ (which is within the bistable region), parameters are presented in table 2.

When the improved crop was tolerant, however, such an ‘all-control’ equilibrium was possible (figure 3*b*). The primary benefit of tolerant crop is that, when infection occurs, there is a lower loss of yield compared with unimproved (or resistant) crop. As there is no reduction in the viral titre, tolerant crops are just as likely to act as sources of infection as unimproved crops. Thus, the benefits of planting tolerant crop are experienced by the grower that plants tolerant crop, so there is no disincentive for other growers to also use it.

We then further investigated the initial conditions that could lead to bistability. Figure 4*a* shows the effect of the rate of horizontal transmission (*β*) on the stability of equilibria. There is a bistable region between *β* = 0.02 and 0.0333 d^−1^, and which equilibrium (either the disease-free or all-control equilibrium) is attained will depend on initial conditions. The discontinuous system leads to kinks in the graphs, whose ordering follows that outlined in electronic supplementary material, appendix 2.

The initial proportions of growers using tolerant crop (*S*_*C*0_ + *E*_*C*0_ + *I*_*C*0_) and infectious fields (*I*_*U*_ + *I*_*C*_) had a large impact on the final proportion of infectious fields. We did this for *β* = *δ*_*β*_*β* = 0.0028 d^−1^, which is within the bistable region in figure 4*a*. At very low levels of both initially controlled and infectious fields, the system always goes to the DFE, with no disease persisting (figure 4*b*). As both increase, disease is more likely to invade until both controlled, and infectious fields are sufficiently high that the system will always go to a disease-endemic equilibrium.

We compared two sets of initial conditions, which differed in their initial proportion of infectious fields. In figure 4*c*, *S*_*C*0_ + *E*_*C*0_ + *I*_*C*0_ = 0.1 (i.e. initially 10% of fields are planted with improved crop). In the ‘high infection’ scenario, 10% of fields are infectious; for the ‘low infection’ scenario, this is just 1%. In the former scenario, disease persisted at equilibrium, whilst it died out in the latter.

The ratio of initially infectious improved and unimproved crop (figure 4*d*) was important in determining disease persistence. If 70% of initially infected fields were tolerant ((*S*_*U*0_, *E*_*U*0_, *I*_*U*0_, *S*_*C*0_, *E*_*C*0_, *I*_*C*0_) = (0.83, 0, 0.03, 0.03, 0, 0.07); ‘high *I*_*C*_’) disease persisted as shown in figure 4*c*. However, if there were fewer infectious tolerant fields ((*S*_*U*0_, *E*_*U*0_, *I*_*U*0_, *S*_*C*0_, *E*_*C*0_, *I*_*C*0_) = (0.83, 0, 0.07, 0.07, 0, 0.03); ‘low *I*_*C*_’), disease died out. Although this is not the only condition for disease persistence when *R*_0_ < 1, as at high levels of *I*_*U*0_, disease can persist even if there are no *I*_*C*0_ fields, these results cumulatively suggest that the presence of infected tolerant crop early in the epidemic can lead to alternative equilibrium being attained. This is possibly driven by the lower probability of detection of tolerant crop (*δ*_*ν*_*ν* = 0.1), which means that it is not removed quickly once it becomes infectious and allows disease to spread.

### 3.3. Q3: Impact of tolerant or resistant crop on grower behaviour

#### 3.3.1. Effect of epidemiological and economic parameters on grower behaviour

**Figure 5.**
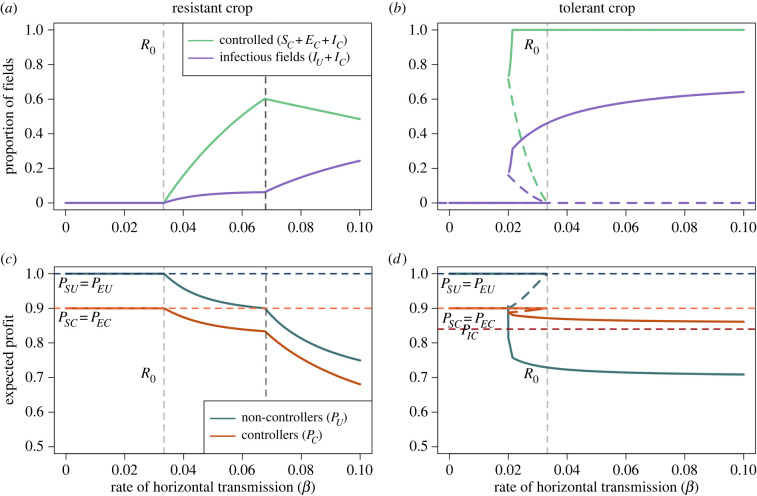
Response to the rate of horizontal transmission in non-improved crops (*β*). (*a*) Change in controllers and infectious fields when the improved crop has resistant characteristics. Generally, with an increase in *β* comes an increase in *C*, though at *β* = 0.067 d^−1^ there is a decrease and corresponding increase in infectious fields. (*b*) The change in proportion of growers using the improved crop (*C*) and infectious fields (*I*_*U*_ + *I*_*C*_) when the improved crop has tolerant characteristics. (*c*) Change in the expected profit for non-controllers (*P*_*U*_, equation app. (11)) and controllers (*P*_*C*_, equation app. (12)) for resistant crop. Until *β* = 0.067 d^−1^, controllers with susceptible or latently infected crop should switch strategy as *P*_*U*_ > *P*_SC,EC_. After this point, they should stop switching. As *P*_ICH,ICR_ < *P*_*U*_, these growers should always change strategy, leading to a fall in the proportion of controllers. (*d*) Change in the expected profit for non-controllers (*P*_*U*_) and controllers (*P*_*C*_). For each value of *β*, growers managing susceptible or latently infected fields of neither strategy should change (as *P*_*U*_ < *P*_SC,EC_ and *P*_*C*_ < *P*_SU,EU_). In (*c*,*d*), the dashed lines show the outcomes achieved by different classes of growers (given by equations (2.9)–(2.16)), which do not change with changing *β*. Other than those being varied, parameters and initial conditions are as in tables 2 and 3, respectively.

We first investigated the impact of changes to the rate of horizontal transmission on the adoption of improved crop. For all values of the rate of horizontal transmission in non-improved crop (*β*), the proportion of infected fields was higher when the improved crop was tolerant than when it was resistant (figure 5*a* vs *b*). Disease could also invade at a lower value of *β* (in the bistable region where *R*_0_ < 1). The expected profits for both strategies were higher when there was resistant crop (figure 5*c*,*d*), and the non-controllers gained more benefit from others using the resistant crop than from the tolerant crop.

Generally, for both crop varieties, as *β* increased so too did the proportion of fields controlling. With the tolerant improved crop, this led to a higher proportion of infectious fields (*I*_*U*_ + *I*_*C*_; figure 5*b*), as tolerant crops have a reduced probability of being rogued, so there is a higher disease pressure on other fields in the system. Once disease invades at no point should growers of either strategy that have susceptible or latently infected fields switch strategy as *P*_*U*_ < *P*_SC,EC_ and *P*_*C*_ < *P*_SU,EU_.

The trend is broadly similar for resistant crop. However, as *β* increases, there is a decrease in the proportion controlling. As *P*_*U*_ approaches *P*_SC,EC_, fewer growers managing fields of these types should switch strategy. However, the increase in infection pressure means that more of these fields will become infected, achieving the lowest payoff. Thus, the growers have a non-zero probability of switching strategy.

Once *β* > 0.067 d^−1^ (marked by the vertical dashed line in figure 5), the proportion of controllers falls. At this point, the high infection pressure means the expected profits of non-controllers exceed those of susceptible or latently infected controllers, so they do not switch strategy (*P*_*U*_ > *P*_SC_ = *P*_EC_). Yet the fields of these controllers are still likely to get infected as resistance is incomplete, so the growers will incur the double penalty of the cost of control and loss due to disease. Thus, as *P*_ICH,ICR_ < *P*_*U*_ for all values of *β*, growers managing infected fields with improved crop should always consider switching strategy. As there are now fewer fields planted with resistant crop and the overall disease pressure increases, so too does the number of infectious fields.

**Figure 6.**
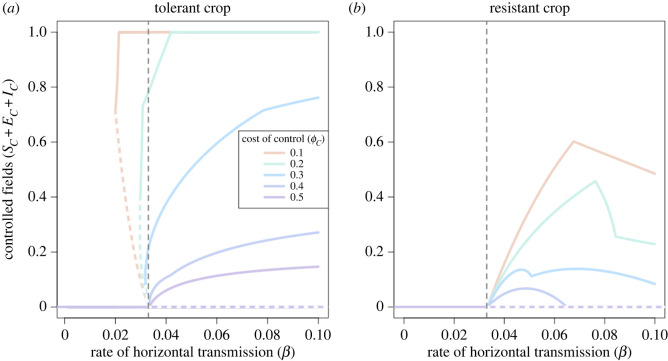
Effect of the cost of control (*ϕ*_*C*_) on uptake of improved crop. The grey dashed lines show where *R*_0_ = 1), and white dashed lines show unstable equilibria. (*a*) When improved crop is tolerant, when *ϕ*_*C*_ ≤ 0.3, bistability is possible. The range of *β* values for which bistability exists is larger with a lower *ϕ*_*C*_. Even at very high costs of control (*ϕ*_*C*_ > 0.4), control persists at equilibrium. (*b*) When improved crop is tolerant, no bistability exists. Only once *R*_0_ > 1 can control persist. However, as *β* gets larger, even at low costs of control, fewer growers use resistant crop. Note, the maximum possible value for *ϕ*_*C*_ when the crop is resistant is *ϕ*_*C*_ = 0.4, otherwise payoff for infected resistant crop, *P*_IUH_, would be negative for the default paramet rization.

The response to infection rate changed for different values of the cost of control, *ϕ*_*C*_ (figure 6). When the parametrization was tolerant (figure 6*a*), bistability existed for values of *ϕ*_*C*_ < =0.3, though the region where bistability was possible narrowed as the cost of control increased. When *ϕ*_*C*_ = 0.1 or 0.2, there existed a scenario where grower behaviour meant that only tolerant crop was possible. Even at very high costs of control (*ϕ*_*C*_ = 0.5), some growers nevertheless controlled at equilibrium.

When the improved crop is resistant, disease can only invade once *R*_0_ > 1, irrespective of the cost of control. Once disease invades, control only persists for a narrow range of *β*, as above a certain threshold, control is seen as too costly (as growers are more likely to pay the dual penalty of the cost of control and loss due to disease, *L*_*C*_, thus earning the lowest possible profit, *P*_ICH_). The range for which resistant crop is used at all is narrower for larger values of *ϕ*_*C*_. The kinks in this graph (such as in figure 6*a* when *ϕ*_*C*_ = 0.3 and *β* = 0.78 d^−1^ or figure 6*b* when *ϕ*_*C*_ = 0.1 and *β* = 0.72 d^−1^) are caused by changes in the switching terms, discussed in detail in electronic supplementary material, appendix 6, figure 1.

#### 3.3.2. Comparison of profits for tolerant and resistant crop

**Figure 7.**
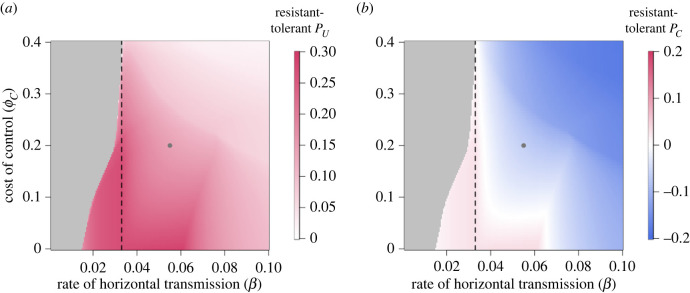
Difference in expected profits when there is resistant crop vs tolerant crop. (*a*) The difference in expected profit for non-controllers (*P*_*U*_) when resistant or tolerant crop is used. (*b*) The difference in expected profit for controllers (*P*_*C*_) when resistant or tolerant crop is used. The grey dots indicate the default paramet rization, and the grey section below *R*_0_ = 1 (the black dashed line) is where there is no difference in profit due to the lack of improved crop being used. Other than those being varied, parameters and initial conditions are presented in tables 2 and 3, respectively.

We now compare the expected profits at equilibrium for controllers and non-controllers when the improved crop is either tolerant or resistant (figure 7, which shows the difference in expected profit for non-controllers (*a*) and controllers (*b*) when crop is resistant and when it is tolerant). The expected profits for unimproved, tolerant and resistant crop are shown in electronic supplementary material, appendix 6, figure 3. When generating these graphs, we chose initial conditions that would always guarantee a disease-endemic equilibrium in the bistable region to ensure that differences are calculated between comparable equilibria (*I*_*U*0_ + *I*_*C*0_ = 0.15, *S*_*C*0_ + *E*_*C*0_ + *I*_*C*0_ = 0.2, figure 4*b*).

Directly comparing the types of improved crop and their effect on the expected profits we can see that for non-controllers, the presence of the improved crop in the system is always more beneficial when crop is resistant (figure 7*a*). However, whether tolerant or resistant crop improves controllers’ profits depends on parameter values (figure 7*b*). At low-to-medium costs of control (*ϕ*_*C*_) and transmission rates (*β*), those growing resistant crop earn slightly more than they would if they used tolerant crop. Both improved crop types cost the same amount (*ϕ*_*C*_), so it is more advantageous to use resistant crop when there is already a low probability of infection, and effectively completely avoid incurring the yield loss due to infection, than to be tolerant and risk losing yield (even if the yield loss itself is small).

In both graphs, the increase and subsequent decrease in the benefit of resistance for non-controllers and resistant controllers that begins when *β* = 0.064 d^−1^ in figure 7*a*,*b* is caused by changes in the switching terms (electronic supplementary material, appendix 2) and changes in infection pressure.

## 4. Discussion

Although tolerance and resistance to disease have been widely researched in plant biology, little thought has been given to the distinct epidemiological consequences of deploying tolerant or resistant varieties upon a community of growers, and none to how this affects the decisions of growers to partake in control. Growers’ behaviour more broadly, and its effects on epidemic outcomes remains largely overlooked in plant disease epidemiology (with some exceptions including [30–33,59–61]). Here, we investigated the effect of a fixed proportion of ‘improved’ crop (with either tolerant or resistant characteristics) on growers’ profits and subsequently how this affected growers’ use of improved crop when given the choice. Although the models we developed can in principle be applied to a broad range of pathosystems, we demonstrate the model using TYLCV as a case study.

As is intuitive from the underlying epidemiology, when a fixed proportion of growers was assigned tolerant crop, there was an increase in the proportion of infectious fields compared with when the improved crop was resistant (figure 2*b* vs *d*). However, for the tolerant crop, the negative impact of having more infectious fields was predominantly felt by the growers using unimproved crop, who saw a larger fall in profits than the controllers. This was due to the negative externalities generated by the use of tolerant crop; as tolerant crop has reduced symptom development, it has a lower rate of removal via roguing. This allows more disease to build in the system, increasing the probability of infection for fields with unimproved crop and consequently reducing the profit of non-controllers.

When there was a constant proportion of growers using each strategy, when the proportion using resistant crop, *C* > 0.55, disease goes extinct (figure 2*d*). Despite the increase in profits experienced by all growers when disease was eliminated via the use of the resistant crop, most of the benefit was experienced by non-controllers, who saw a more substantial increase in profits than the controllers (figure 2*b*). This echoes past studies showing that if some proportion of growers in a landscape do control for disease, the benefits are widely felt [9,19,22]. In our case, growers of resistant crop generated positive externalities, benefiting others whilst incurring a cost themselves. Thus, the resistant crop is more beneficial to growers of both strategies than tolerant crop (which cannot be used to eliminate disease and increase the payoffs of non-controllers).

The nature of these contrasting externalities suggests that, given the choice, relatively few growers should choose to use resistant crop when available as they will gain more benefit when they do not (i.e. they will ‘free-ride’ off of the costs incurred by controllers). Similarly, it suggests growers using tolerant crops incentivizes others to do so too, as they will have higher yields when infected. To investigate these dynamics, we included growers’ behaviour in our disease spread model, with growers evaluating profitability based on the ‘grower vs alternative strategy’ method described in [32] (which takes the same form as decision models in [30,31,33]). In this model, growers compared their own outcome from the previous season (i.e. whether they had a rogued field that had resistant crop, a susceptible field with unimproved crop) with the average expected profit of the alternative strategy. The model is based on ‘strategic-adaptive’ expectations, which balance a grower’s previous experience against the probability of future events [62].

Once growers’ behaviour is introduced, the threshold proportion of growers using resistant crop needed to eliminate disease is never reached, even at high disease pressures (figure 3*a*). This is because of ‘free-riding’; growers gain more benefit from the protection provided by others using resistant crop than they would if they themselves used resistant crop (in figure 5*c*, the expected profit for non-controllers is higher than for controllers (*P*_*U*_ > *P*_*C*_)). Thus, although the use of resistant crop can theoretically lead to disease elimination, when considering the behaviour of growers it is not possible.

When the improved crop was tolerant, however, bistability between disease-free and disease-endemic equilibria was observed when the basic reproduction number (*R*_0_) was less than unity (figure 3*b*). Previous epidemiological models including factors such as imperfect vaccination, risk-structure or re-infection (e.g. [63,64], summarized in [58]), vector dynamics [65,66], fungicide application [67] or aspects of individual behaviour [68,69] have also identified such bistable regions. In our case, changing the rate of horizontal transmission (*β* = *δ*_*β*_*β* for the tolerant parametrization) induced this bistability, and whether the system went to a disease-free or disease-endemic equilibrium depended on the initial proportion of infectious fields and initial proportion of controllers (figure 4*b*). Increasing the cost of control reduced the size of the region in parameter space in which bistability was observed (figure 6*a*). If the cost of control is sufficiently high such that the tolerant crop is never more profitable than unimproved crop, the bistable region is eliminated. Bistability was not observed when the improved crop had the default resistant parametrization (figures 3*a* and 6*b*). Thus, the use of tolerant crop may lead to less predictable outcomes at lower values of *δ*_*β*_*β*, as having a *R*_0_ < 1 is no longer sufficient to prevent disease spread.

When growers’ behaviour was introduced, an ‘all-control’ equilibrium, where all growers used improved crop, was attained at low rates of horizontal transmission for the tolerant parametrization (figure 5*b*). As seen for the fixed proportions, however, this was accompanied by a higher number of infectious fields and correspondingly low expected profit for non-controllers (figure 5*a*). It is these low expected profits for non-controllers that drive the higher participation in control, as the profits for those using tolerant crop remain high irrespective of the infection pressure. The benefits of using tolerant crop are felt privately by those using it, generating negative externalities for others.

Conversely, such an ‘all-control’ equilibrium was not achieved when the improved crop was resistant, since it generates positive externalities for non-controllers (figures 3*a* and 5*b*). This is a product of both how the model was set up and the nature of the externalities produced. As the lowest possible payoff was achieved by growers of resistant crop that did not rogue their fields (*P*_ICH_), any of these growers should always have a non-zero probability of switching strategy (*z*_ICH_ > 0). Thus, there will always be non-controllers at equilibrium. In addition, the reduced probability of infection meant that the need to control was reduced, disincentivizing growers from switching to the costly control strategy. This conflict between private and social benefits is often observed in epi-economic models and is theorized to be the reason why many vaccination schemes fail to achieve a socially optimal level of vaccination [16,70,71].

Interestingly, despite the positive feedback loop induced by the use of tolerant crop, a mixed ‘unimproved and tolerant crop’ equilibrium was possible. In this equilibrium, even though the growers of tolerant crop should all switch strategy (*P*_*U*_ > *P*_SC,EC,ICR,ICH_), the growers of unimproved crop with infectious fields should also switch strategy (*P*_*C*_ > *P*_IUH,IUR_). Growers of tolerant crop switch strategy because, for all combinations of pairs of values of *β* and *ϕ*_*C*_ which corresponds to a mixed equilibrium in figure 3, *P*_*U*_ (the profit for unimproved crop) is greater than the profits for at least one of the outcomes that can be attained by a grower of the tolerant variety. At the higher costs of control, *P*_*U*_ > *P*_SC_, *P*_EC_, *P*_ICR_ and *P*_ICH_. However, for all regions of the mixed equilibrium, at least one group of tolerant growers should be switching strategies (i.e. one of equations (16)–(18) in Scenario (i) of electronic supplementary material, appendix 2 should be true). This causes a flow between the two strategies—tolerant and unimproved—and this in turn leads to a mixed equilibrium. The existence of a mixed equilibrium is not critically dependent on the parametrization adopted for figure 3, and a mixed equilibrium is possible for parametrizations corresponding to the other scenarios identified in electronic supplementary material, appendix 2. In particular, the flow between tolerant and unimproved can also be found in the following cases: equations (22)–(25) of Scenario (ii), equations (28)–(30) of Scenario (iii) and equations (34)–(36) in Scenario (iv). So long as there are flows between both strategies, a mixed tolerant and unimproved equilibrium is possible and is attained within our model.

For growers who choose to use improved crop, tolerant varieties generally give better outcomes than resistant ones (except in cases where infection is unlikely enough that the probability of incurring the loss due to disease is low) (figure 7*b*). This will generate lower payoffs for non-controllers, who earn higher profits when there are more resistant fields (figure 7*a*). In our model, there was widespread use of control when the tolerant crop was effective at reducing yield loss and not too costly as to discourage control when the probability of infection is low. However, this came at the cost of increasing the level of infection in the system and reducing the profits for non-controllers.

Several simplifying assumptions were made during this investigation. Our model was deterministic and did not account for spatial effects, both of which can influence epidemic outcomes. In our behavioural model, we assumed that all growers would have access to the same information regarding disease pressure and the expected profits. In reality, a grower’s knowledge of these quantities will be highly dependent on their communication network [31], their trust in expert knowledge [72], their experience with previous outbreaks [73] etc. How growers react to differences in profit (represented by our parameter *η*) will also vary between individual growers and will impact long-term outcomes [32]. Growers must balance these information sources with market demands to make their decisions regarding disease control, and become what Kaup terms the ‘reflexive producer’ [74].

Overall, this study has shown that tolerant and resistant varieties of crop have different effects on disease outcomes and provide benefits to different groups of growers (controllers vs non-controllers). In particular, even when resistant crop was available, disease was never eliminated from the system (even though it was theoretically possible) as too few growers chose to use the resistant variety. This is an important consideration, as previous studies have found optimal cropping ratios for different sets of conditions (e.g. [75]); in reality, the strategic decision making of growers, as well as other factors such as their access to information or risk aversion, may mean that these ratios are never attained. Accounting for these behaviours can help improve future models of control uptake and in turn our understanding of how plant diseases spread.

## Data Availability

Base code is available at: https://github.com/RachelMurray-Watson/Tolerant-crops-increase-growers-yields-but-promote-selfishness-how-the-epidemiology-of-disease-res. The data are provided in electronic supplementary material [76].
